# A Text-Messaging and Pedometer Program to Promote Physical Activity in People at High Risk of Type 2 Diabetes: The Development of the PROPELS Follow-On Support Program

**DOI:** 10.2196/mhealth.5026

**Published:** 2015-12-15

**Authors:** Katie Morton, Stephen Sutton, Wendy Hardeman, Jacqui Troughton, Tom Yates, Simon Griffin, Melanie Davies, Kamlesh Khunti, Helen Eborall

**Affiliations:** ^1^ UKCRC Centre for Diet and Activity Research (CEDAR) MRC Epidemiology Unit University of Cambridge Cambridge United Kingdom; ^2^ Behavioural Science Group, Primary Care Unit Department of Public Health and Primary Care University of Cambridge Cambridge United Kingdom; ^3^ School of Health Sciences University of East Anglia Norwich United Kingdom; ^4^ Leicester Diabetes Centre University Hospitals of Leicester National Health Service Trust Leicester United Kingdom; ^5^ Diabetes Research Centre College of Medicine, Biological Sciences and Psychology University of Leicester Leicester United Kingdom; ^6^ NIHR Leicester-Loughborough Diet, Lifestyle, and Physical Activity Biomedical Research Unit Leicester United Kingdom; ^7^ Primary Care Unit Department of Public Health and Primary Care University of Cambridge Cambridge United Kingdom; ^8^ NIHR Collaboration for Leadership in Applied Health Research and Care - East Midlands (NIHR CLAHRC- EM) East Midlands United Kingdom; ^9^ Social Science Applied to Healthcare Improvement Research Group Department of Health Sciences University of Leicester Leicester United Kingdom

**Keywords:** physical activity, mHealth, text messaging, pedometer, tailoring, type 2 diabetes, intervention development

## Abstract

**Background:**

Mobile technologies for health (mHealth) represent a promising strategy for reducing type 2 diabetes (T2DM) risk. The PROPELS trial investigates whether structured group-based education alone or supplemented with a follow-on support program combining self-monitoring with pedometers and tailored text-messaging is effective in promoting and maintaining physical activity among people at high risk of T2DM.

**Objective:**

This paper describes the iterative development of the PROPELS follow-on support program and presents evidence on its acceptability and feasibility.

**Methods:**

We used a modified mHealth development framework with four phases: (1) conceptualization of the follow-on support program using theory and evidence, (2) formative research including focus groups (n=15, ages 39-79 years), (3) pre-testing focus groups using a think aloud protocol (n=20, ages 52-78 years), and (4) piloting (n=11). Analysis was informed by the constant comparative approach, with findings from each phase informing subsequent phases.

**Results:**

The first three phases informed the structure, nature, and content of the follow-on support program, including the frequency of text messages, the need for tailored content and two-way interaction, the importance of motivational messages based on encouragement and reinforcement of affective benefits (eg, enjoyment) with minimal messages about weight and T2DM risk, and the need for appropriate language. The refined program is personalized and tailored to the individual’s perceived confidence, previous activity levels, and physical activity goals. The pilot phase indicated that the program appeared to fit well with everyday routines and was easy to use by older adults.

**Conclusions:**

We developed a feasible and innovative text messaging and pedometer program based on evidence and behavior change theory and grounded in the experiences, views, and needs of people at high diabetes risk. A large scale trial is testing the effectiveness of this 4-year program over and above structured group education alone.

**Trial Registration:**

International Standard Randomized Controlled Trial Number (ISRCTN): 83465245; http://www.controlled-trials.com/ISRCTN83465245/83465245 (Archived by WebCite at http://www.webcitation.org/6dfSmrVAe)

## Introduction

### Background

Like most developed countries, the United Kingdom is facing a growing prevalence of type 2 diabetes (T2DM) [1]. Furthermore, in England there has been a marked increase in the number of people identified with impaired glucose regulation (IGR): blood glucose levels higher than normal but below the threshold for T2DM and associated with increased risk of developing T2DM and further complications [2]. Given the significant economic burden of treating T2DM [3], prevention of the condition is a public health priority.

The main targets for T2DM prevention are weight loss and physical activity promotion [4,5]. Physical activity slows the progression of T2DM and its cardiovascular consequences [6] and thus is often argued to be a cornerstone of T2DM prevention initiatives [5]. Indeed, several large, high-quality clinical trials have shown that relatively modest changes in lifestyle (eg, increased physical activity) can reduce its incidence [7,8].

Structured self-management education is recommended for facilitating lifestyle change (including physical activity) among people with T2DM and those identified as being at high risk of developing T2DM [9]. The Pre-diabetes Risk Education and Physical Activity Recommendation and Encouragement (PREPARE) study, which combined group-based structured education and pedometer use, reported improvements in glucose regulation in people at high risk of T2DM [10]. Notably, only the group that received a pedometer in addition to structured education demonstrated better clinical outcomes. Indeed, meta-analyses have shown that interventions that prompt self-monitoring by pedometers resulted in increased physical activity [11,12]; among individuals with T2DM, walking programs that do this have shown that they are feasible and effective at increasing moderate intensity bouts of physical activity [13,14].

T2DM prevention guidelines recommend the provision of ongoing support for people identified as being at risk, particularly when barriers for behavior change are encountered [9,15]. Although primary care offers a system for identifying individuals at high risk of T2DM (eg, through the National Health Service [NHS] Health Checks in England), it lacks the capacity and resources to offer ongoing support to the patient through regular face-to-face contact with health care professionals. As such, there is a need to develop and evaluate scalable and cost-effective T2DM prevention programs that provide ongoing behavior change support beyond structured education and pedometers and are suitable for implementation in routine care [16]. Tailored, computer-generated feedback on pedometer-measured step counts may be a cost-effective way to provide ongoing support for physical activity among people at high risk for T2DM. One way of achieving this is through the use of mHealth (ie, mobile phone technology [17]), specifically via short message service, hereafter referred to as “text messaging.”

### mHealth Approaches

While smartphone ownership is increasing (estimated at 55% in the UK adult population), it is less than 20% in people aged 65 years and older who are more likely to be at risk of T2DM [18]. Nonsmart mobile phone ownership, estimated at 77% in those aged 65-74 years [19], is commonplace; hence, text messaging has a potentially wider reach in this group. Furthermore, text messaging can be automated and individually tailored, and it allows frequent delivery with asynchronous receipt (ie, people can choose when to read the messages). Thus, it is potentially an efficient delivery channel for providing participants with information, feedback, and a choice of when to access messages.

Text-messaging interventions are increasingly used in T2DM prevention. A recent randomized controlled trial (RCT) [20] evaluated a text-messaging T2DM prevention intervention delivering randomly generated lifestyle advice messages to men aged 35-55 years in India. It reported significantly lowered incidence of T2DM at 24-month follow-up. However, no between-group differences in self-reported physical activity were observed. A T2DM prevention intervention in a general population (mean age of 42 years) [21,22] that sent very frequent (5-7 per week) tailored messages (including general educational messages, diet and exercise tips, and health reminders) and prompt messages to encourage goal setting increased participant risk awareness and knowledge of T2DM. However, the majority of text-messaging interventions for T2DM self-management and prevention have targeted clinical outcomes only, in younger and middle-aged adults (55 years and younger), and have not measured behavioral outcomes (eg, physical activity) [23].

It is widely accepted that the development of complex behavior change interventions, including mHealth approaches, should be informed by behavior change theory, evidence, and formative research [24,25] and that sufficient details of the final intervention are reported [26,27]. Yet, many published mHealth studies of physical activity promotion do not describe the structure, content, or evidence base for the intervention in enough detail to allow replication. Taken together, there is uncertainty about the active ingredients, effectiveness, feasibility, and acceptability of evidence-based mHealth to increase physical activity in a population of people at risk of T2DM which includes older adults.

### Context: Walking Away From Diabetes and the PROPELS Trial

Walking Away from Type 2 Diabetes [28,29] is an annual group-based, structured education session (hereafter referred to as “Walking Away”). It is typically delivered to groups of 4-10 individuals by 2 trained educators over 3 hours. It is designed to promote walking by targeting perceptions and knowledge about IGR and physical activity self-efficacy as well as promoting self-regulatory skills such as goal setting, self-monitoring, and problem solving for relapse prevention. Participants receive a pedometer, but there is no additional contact with educators beyond the session and hence no feedback on individual progress.

PROPELS (Promotion Of Physical activity through structured Education with differing Levels of ongoing Support for those at high risk of type 2 diabetes) [ISRCTN83465245] is a multisite RCT that aims to examine the long-term effectiveness of the Walking Away education with different levels of ongoing support (over 4 years) [30]. The RCT includes 3 arms: Group 1 receives an informational advice leaflet; Group 2 receives the leaflet, annual Walking Away sessions, and a pedometer; and Group 3 receives the leaflet, annual Walking away sessions, and pedometer plus a comprehensive follow-on support program using pedometer self-monitoring, tailored text messaging, and telephone calls.

### Purpose

This paper describes the iterative development of the PROPELS follow-on support program and presents evidence about its feasibility and acceptability. The protocol for the PROPELS RCT is published elsewhere [30].

## Methods

### Design and Framework

**Figure 1:**
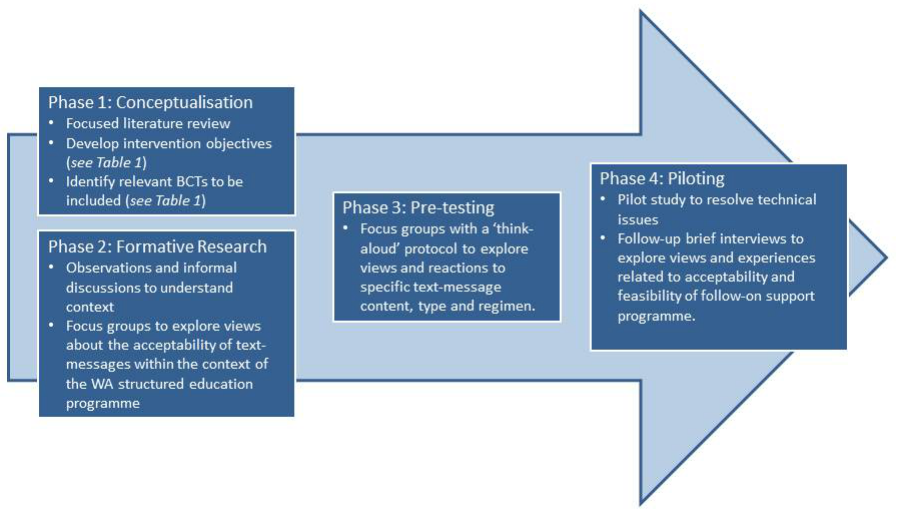
Design and framework of the PROPELS follow-on support program.

To develop the PROPELS follow-on support program, we used a structured, iterative process involving concurrent and sequential research with the target population while maintaining a strong focus on integration of theory and evidence. Our framework for intervention development and piloting was informed by the model by Dijkstra and De Vries [31] for developing computer-generated tailored interventions (to conceptualize the program) and the mHealth development and evaluation framework by Whittaker et al (2012) [32] and Fjeldsoe et al (2012) [33]. An outline of our framework is shown in Figure 1. This development study was approved by National Research Ethics Service Committee East Midlands-Leicester (12/EM/0151) as part of the PROPELS RCT.

### Phase 1: Conceptualization

We conducted a focused literature review to identify the key psychosocial determinants of increasing and/or maintaining physical activity levels among adults at risk of developing T2DM (see Phase 1 results for the key findings from this review). In line with Dijkstra and De Vries’ [31] model of developing computer-generated tailored interventions, we then translated these determinants of physical activity into the key objectives (see Multimedia Appendix 1) of the PROPELS follow-on support program.

### Phase 2: Formative Research

In parallel with Phase 1, we (KM and HE) conducted informal observations of Walking Away sessions in diverse regions where it has been commissioned into routine care pathways for the prevention of T2DM. In addition, we (KM, HE, and WH) engaged in discussions with Walking Away educators involved in an ongoing evaluation of Walking Away taking place within primary care [29]. In this phase, we aimed to become familiar with the delivery of Walking Away, develop initial ideas about the PROPELS follow-on support structure and content, understand the cultural and ethnic diversity of our target population, explore educators’ views about supplementing Walking Away with text messaging and pedometer support, and inform the development of topic guides for subsequent focus groups.

Following this, we conducted 3 formative focus groups with our target population. Eligibility criteria included having attended the Walking Away session within the last 3 years as part of an ongoing evaluation in primary care [29], having provided consent to be contacted with regard to other research within the department, and having ability to speak and understand spoken English. Potential participants were sent an information leaflet and opt-in reply slip. A researcher telephoned those who had expressed an interest in taking part to check willingness and arrange attendance at a focus group. Written informed consent was taken immediately before the focus groups. A total of 15 participants (5 women and 10 men) aged between 39 and 76 years participated. A flexible topic guide was used that covered experiences of Walking Away (eg, what was most and least helpful for increasing physical activity and what could be improved to facilitate sustained changes), use of mobile phones in everyday life, and integration of a text-messaging follow-on support program into Walking Away.

Focus groups were audio recorded and transcribed verbatim. Our analytical approach was based on the constant comparative method [34]. Specifically, KM familiarized herself with the data and identified initial codes. This involved organizing the data into meaningful groups and identifying interesting aspects in the data that formed the basis of repeated patterns (themes) across the dataset. Codes were assembled into an initial coding framework (KM and HE); this was used to code the complete dataset. NVivo qualitative data indexing software (QSR International) was used to facilitate the analysis.

### Phase 3: Pretesting

We created exemplar text messages based on the findings of Phases 1 and 2 (see the “Phase 1 and 2 Results” sections) and conducted 4 further focus groups (n=20; ages 52-77). Eligibility was the same as in Phase 2, but we also invited participants from the Walking Away study control group who had not previously attended the program [29]; recruitment and consent procedures were identical to Phase 2. Prior to attending a focus group, participants received a pedometer and activity diary via postal mail and were encouraged to record the number of steps per day for 1 week. Participants were asked to bring along a mobile phone to the focus group.

**Figure 2:**
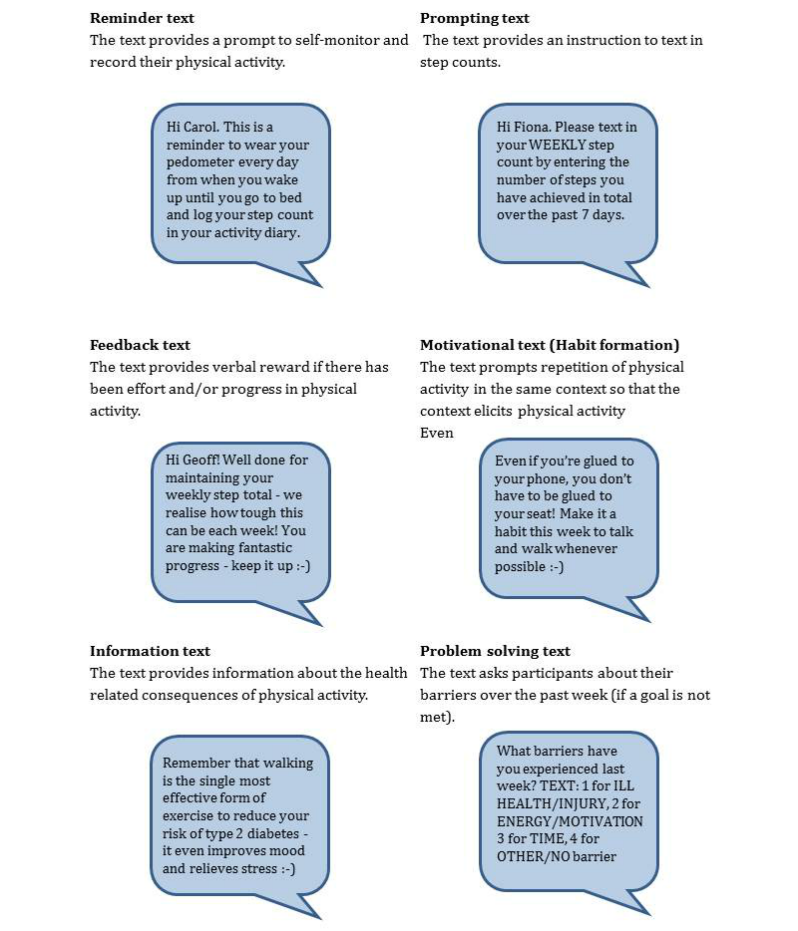
Example text messages used in Phase 3.

As in Phase 2, a topic guide covered experiences of Walking Away. Additionally, it explored experiences of wearing the pedometer and recording steps. During the focus group, participants were sent example text messages (Figure 2) to provoke reactions in situ and generate think-aloud [35] reactions and discussions about different types of messages. Data analyses followed the approach used in Phase 2. The coding framework was further developed from the Phase 2 coding framework to reflect the current phase of development.

### Phase 4: Piloting

Using the findings of Phases 1 through 3, KM drafted an initial set of text messages and tailoring matrices. The tailoring matrices (for each week of the program) specify the individual characteristics to which each message will be adapted. SS developed a computer program to automatically generate and send text messages (in line with the tailoring matrices) and to handle incoming messages. We subsequently tested the content and schedule of the text-messaging and pedometer program and the delivery processes required (eg, registering with the text message system, gathering information for tailoring, and receiving and replying to the messages). We also aimed to identify and resolve potential technical issues with the automated system.

Participants were 11 people (6 men and 5 women) from the Phase 2 and 3 focus groups who had indicated interest, including participants who were less keen on the use of text messages. This 8-week pilot study mimicked the proposed initial 8 weeks of the PROPELS follow-on support program. Participants were mailed an instruction booklet with details on how to register and what to expect from the text-messaging system, a pedometer, and an activity diary. They were instructed to wear the pedometer and self-monitor steps using the activity diary for 1 week to determine a baseline number of steps that would inform their step goals for the next 8 weeks. KM administered the brief telephone assessment to elicit each participant’s short- and long-term step goals, an action plan for increasing physical activity, and information for the tailoring variables. Then, each week, participants received a reminder message to prompt them to submit their weekly step count via text message. This triggered an automated, tailored feedback message with the content depending on goal progress. Participants also received tailored motivational messages if they did not make progress with step counts or text in a step count.

After the 8-week period, KM conducted brief, semistructured telephone interviews with all available participants (n=10) to gain their feedback on the program. Interviews were recorded, transcribed, and analyzed as in Phases 2 and 3.

## Results

### Phase 1: Conceptualization

We focused our literature review on text-messaging interventions to promote physical activity but also reviewed physical activity behavior change interventions within our target population more broadly to identify a number of salient behavior change techniques (BCTs) [36] to incorporate within the PROPELS follow-on support program. The main findings from our focused literature review are as follows.

#### Text Messaging for Physical Activity Promotion

The evidence base for text-messaging interventions to promote health is growing, as demonstrated by two comprehensive meta-analyses. In one meta-analysis that focused on physical activity promotion using mobile devices [37], most of the included interventions delivered through text messaging were passive, sending participants relay messages (eg, goal intentions) or generic, nontailored information about health benefits, and participants were mostly younger adults. There were two exceptions: a pilot study with older people with chronic obstructive pulmonary disease [38] provided the control (self-monitoring) group with a pedometer and mobile phone, prompted them to text in details about their symptoms and exercise, and responded with a standard message to thank them and encourage continued submission of data. Intervention (coaching) group participants received additional ongoing reinforcement coaching messages. Objectively measured step count increased in the self-monitoring group only. The intervention was feasible to deliver; however, delivery was not automated as a nurse manually adjusted text responses, and scalability was limited due to all participants being provided with a phone. The second study, an RCT of a fully automated intervention consisting of a wrist-worn device, an interactive website to provide feedback on physical activity, and text-messaging reminders of activity plans in middle-aged healthy adults reported significant increases in objectively measured activity compared to no support [39].

A second meta-analysis investigated the efficacy of different formats of text-messaging-based interventions for various health behaviors and outcomes. Message tailoring and personalization were significantly associated with greater intervention efficacy [40]. Furthermore, interventions that involved decreasing frequency of messages over the course of the intervention were more effective than interventions that used a fixed message frequency [40]. Text message-only physical activity interventions without tailored feedback did not increase physical activity [41]. Hence, tailored feedback appears to be a promising component of mHealth physical activity interventions.

Taken together, physical activity interventions using text messages may be more effective if they incorporate active components such as self-monitoring, provide tailored feedback and personalized messages, and decrease the frequency of text messages over time.

#### Theory and Behavior Change Techniques Informing the PROPELS Follow-On Support Program

**Figure 3:**
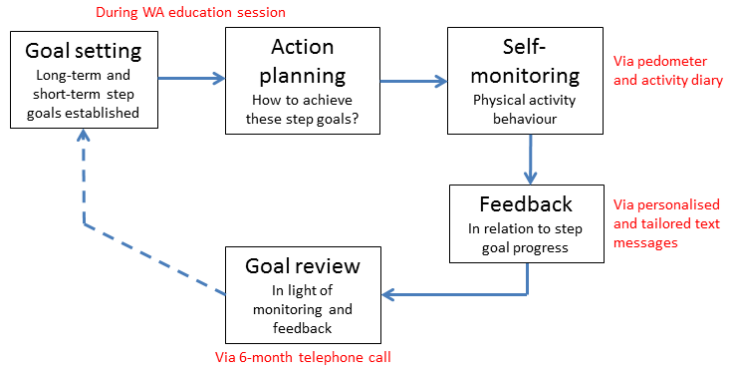
Modified self-regulation control theory which informed the PROPELS follow-on support program.

**Figure 4:**
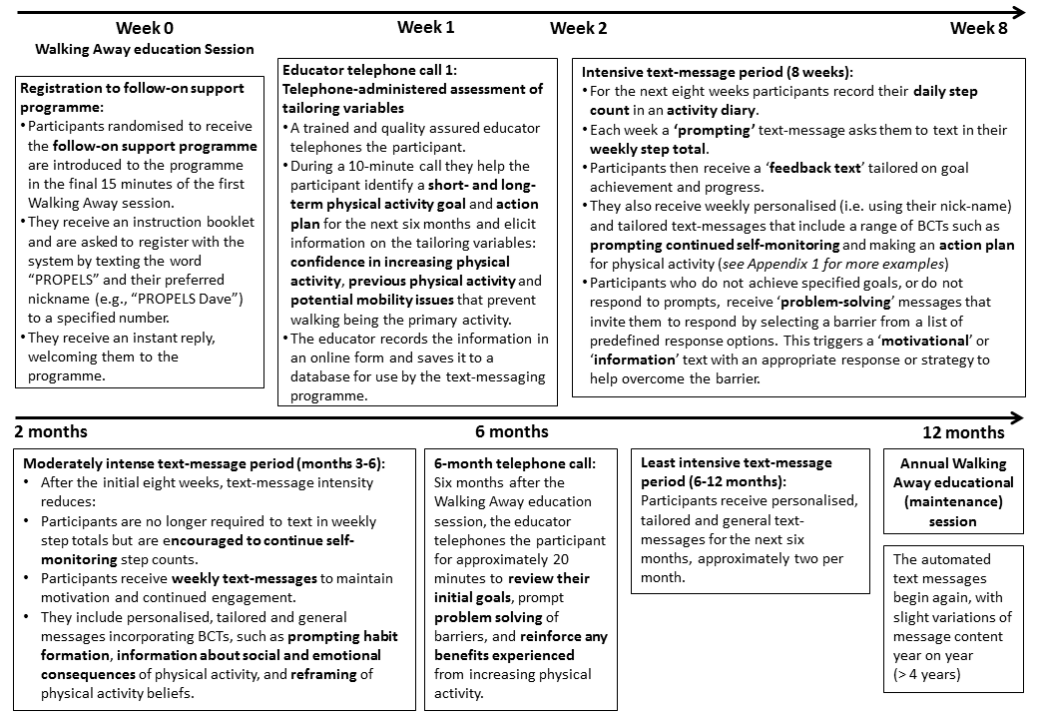
The final PROPELS follow-on support program overview.

Health behavior change interventions (ie, not just text-messaging interventions) that combine self-monitoring with at least one other self-regulatory BCT (eg, goal setting) have been shown to be significantly more effective at increasing physical activity than those that did not include these specific BCTs [42]. These BCTs are congruent with the process of self-regulation or more specifically, control theory [43], which proposes that setting goals, self-monitoring behavior, receiving feedback, and reviewing goals following feedback are central to behavioral self-management. The PROPELS follow-on support program was thus structured around behavioral self-regulation (Figure 3). This facilitated the selection and sequencing of the primary BCTs that are prevalent in the program’s components [36]. Specifically, during the Week 1 educator telephone call, physical activity goals and an action plan were established (Figure 4). The subsequent text-messaging component drew upon a selection of BCTs to (1) encourage self-monitoring of physical activity behavior, (2) provide tailored feedback regarding physical activity progress (to highlight the discrepancy between goals and current behavior), and (3) review behavioral goals. A more detailed description of all BCTs employed within the PROPELS follow-on support program is shown in Multimedia Appendix 1.

Interventions among people with or at risk of T2DM that included a higher number of BCTs [44] or a higher number of BCTs and specific BCTs such as goal setting [45] have been associated with more weight loss. Furthermore, there is consistent evidence demonstrating the importance of several other key determinants of physical activity behavior change across general populations as well as in high-risk groups. These include attitudes toward physical activity [46], intrinsic motivation [47], and (maintenance) self-efficacy [48], especially when this is targeted in conjunction with self-regulation [49]. With this in mind, the PROPELS follow-on support program also targeted other determinants of physical activity behavior change via the text message component and employed additional BCTs to achieve the overall intervention objectives (see Multimedia Appendix 1). Given that uncertainty remains about the acceptability of the aforementioned BCTs when delivered by text message, one aim of Phases 2-4 was to explore the acceptability and feasibility of this approach with our target population.

### Phase 2: Formative Research

The key findings from Phase 2 that influenced intervention development and subsequent phases are presented under two interlinked themes: acceptability of text messaging for physical activity promotion and requirements for the structure of the follow-on program that includes text message content.

#### Acceptability of Text Messaging for Physical Activity Promotion

The majority of participants reported using mobile phones in daily life and being able and willing to use text messages, even if they were not in the habit of doing so as a primary means of communication. Most agreed that text messages could serve as a useful reminder to aid habit formation and provide additional support following an education session.

I think if texting had been in it [Walking Away trial] before it would have helped my motivation a lot.

FG3

The potential ease of integrating a text-messaging program into daily life was highlighted; participants reported that a positive feature was the freedom to choose when to read a message and whether to act on the information provided within it.

...whereas texting is ideal. You can carry on with your normal day-to-day living but still get the motivation.

FG1-A

You don’t have to listen to it, but it’s an idea. You can read all these and just take from it what you want to, don’t you? That’s what we do, gather the information and decide what you want to do from there.

FG3

Another perceived benefit was the opportunity to receive immediate feedback. Many participants reported that a two-way interaction, especially the process of reporting weekly step counts and receiving subsequent feedback, would facilitate motivation and maintenance and could foster a sense of accountability (ie, someone to report to).

It would be good knowing that we’d put the figures in at the end of the week, that you have received them and that you’ve looked at them and that you’re interested in what we’re doing.

FG2-A

Positive views were not unanimous. Some participants felt that texting was “not for [their] generation,” although this did not necessarily mean they were against it.

Well, you know, I think it's just that I don't use it, you know, it's not that I don't like it.

FG1-B

A small number of participants expressed a strong dislike of text messages, reporting that they are intrusive and/or impersonal.

No, I wouldn't [want to receive text messages], I would find that intrusive. It’s bad enough ‘have you been missold PPI,’ ‘have you done this’...so you don't even look at your text messages. If it's not from family I block the lot so no, I wouldn't want text messages.

FG1-C

#### Requirements for the PROPELS Follow-On Support Program

Monitoring and feedback were salient themes. When reflecting on experiences of Walking Away, participants generally reported that the pedometer was a useful monitoring tool that promoted awareness of activity levels.

And it does encourage you because you think, I’ve hardly moved! I think it keeps it in your mind.

FG2

Some participants reported that they were still using it to monitor their physical activity 2-3 years after Walking Away, but the majority reported a lack of continued engagement with the pedometer or activity diary following an initial period of active engagement.

You get up at half six in the morning, you think I’ll go and get a wash and you get changed, and then you go off to work and, “Oh, I didn’t put it on”...you start to forget about it.

FG2-C

Once I've got home I think, oh, I don't think I’ll do any more, I sit on the computer or watch the telly, I need someone to push me out, get out the chair and go and do a walk.

FG3-C

Closely tied to the notion of self-monitoring was the importance of feedback for facilitating behavior change and maintenance. Participants commonly reflected that a lack of contact between the annual Walking Away group education sessions had decreased their motivation to continue with the strategies discussed in the session (eg, setting goals, wearing a pedometer). Several participants described how feedback on their goal setting and progress with increasing physical activity levels would have been useful.

It would have been nice to have the results of that [physical activity measures] because we never knew about that.

FG1-D

The preferred content of the text messages differed greatly according to individual preferences and characteristics. Some participants, especially those who described themselves as self-motivated and the sporty-type (ie, someone who has been fairly active in the past) wanted very different messages from those who described themselves as sedentary and needing more of a push. Furthermore, several participants reported prominent mobility issues, such as osteoarthritis, which meant that content focusing solely on walking was not relevant to them. Hence, the idea of the follow-on support content being tailored to individual characteristics (see Phase 3 for more detail) was appealing to participants.

Participants were adamant that text messaging should supplement, rather than replace, face-to-face contact, especially in relation to strengthening motivation. Some suggested that telephone support, in addition to text messages, could foster rapport between PROPELS educators and participants and provide additional support that cannot be communicated via a text message, thus overcoming the perception that text messages are impersonal.

But, [if] you've got somebody there you can speak to...say, ‘right I'm having a problem, I've done such and such and I can’t register me steps’ or whatever, it's just about [the educator] saying ‘right, you should do this’ or ‘I’ll get somebody to ring you back and tell you what to do,’ you can't do that on text can you?

FG1-E

Taken together, the Phase 2 findings indicate the need for (1) two-way interaction (ie, inputting of step counts and providing immediate feedback about physical activity progress), (2) timely reminders for self-monitoring of physical activity, (3) further consideration of how to overcome perceived barriers to using text messaging (ie, by providing participants with an overview of the benefits of text messaging for follow-on support at the initial Walking Away session), (4) tailored and personalized text message content (explored further in Phase 3), and (5) additional telephone support to enhance rapport between educator and participant and provide support beyond text messages only (ie, problem solving and in-depth social support).

### Phase 3: Pretesting

During the pretesting focus groups, participants were sent a variety of text-messages developed as a result of Phases 1 and 2. Messages included “reminder texts,” reminders to wear the pedometer and log daily steps and instructions to text in step counts; “feedback texts,” feedback about behavior including social reward and positive reinforcement; “motivational texts,” messages using BCTs to strengthen motivation for physical activity (eg, habit formation, commitment, reframing physical activity beliefs); “information texts,” information about health consequences; and “problem-solving texts,” which contained response options to a list of predefined barriers when a goal was not met (Figure 2). Depending on a participant’s response to the latter, they were sent a tailored motivational or information text.

We present key themes emerging from the Phase 3 pretesting focus groups that informed the final content of the PROPELS follow-on support program. We categorize the data into views about self-monitoring of physical activity and text message type, language, and frequency.

#### Self-Monitoring of Physical Activity

The majority of participants reported that self-monitoring their daily steps with the pedometer increased their motivation to be more active due to increased awareness of their own activity.

I found the pedometer really, really useful. I didn’t wear it all the time, but once I wear it I make sure I do 10,000 steps. If I looked at it half way through the day and think I’ve only done 5,000 then I went out for a walk purposely just to get the figures up.

FG4

Some participants found the pedometer demotivating or disheartening, especially those with mobility problems who felt that because they could not engage in walking as their primary activity, the step count was always low.

I wish that it was not just dependent on the steps. Because we do all sorts of other things rather than just steps.

FG7

For these individuals, the self-monitoring process should allow for other activities to be counted (eg, swimming, gardening).

#### Text Message Type, Language, and Frequency

##### Reminder Texts

Several participants commented that establishing appropriate frequency of reminder texts was key to avoiding the intervention becoming off-putting and Big Brother-like or “checking up on you,” especially when people had developed a habit of wearing the pedometer. This suggested a reduction in reminders as the intervention progresses.

If you’ve got something constantly...well, not constantly, but weekly reminding you to do something then you’re still there doing it. And possibly if you’re doing it for several weeks then you’ll get actually used to wearing it and putting it on. It’s like putting your clothes on. You put your socks on, put your pants on, “oh I’ll put my thing [pedometer] on.” It’s all getting used to what you’re doing, like with your lifestyle.

FG5

##### Prompting Texts

Participants were generally happy with the idea that a text message would prompt them to input their weekly step count; this was considered a useful motivational tool.

I suppose the very fact that we would be doing it [texting in step counts] we are creating a certain level of discipline which we didn’t have before.

FG4

##### Feedback Texts

The exemplar feedback messages for having achieved one’s step goal (ie, positive reinforcement) were well received, again fostering a sense of accountability.

We are all school kids in a sense, in our heads, so if someone says you did well it’s really encouraging

FG5

We tested a variety of feedback messages for the event of not achieving one’s step goal. The consensus was that these should be fairly light-hearted, positive, and encouraging. Messages that emphasized a discrepancy between the person’s current behavior and goal were well received, as long as the texts also offered encouragement and support, for example, by including positive elements along with more negative feedback.

...you’ve got to put in, you know, the positive that eliminates some of the negativity out of the messages. So this one was ‘thanks for the text, keep wearing your monitor and logging your steps, try to increase your activity to ensure’...it’s not quite positive enough.

FG4

Indeed, several participants commented that humor could be used to provide feedback when not achieving a step goal.

You can't castigate somebody but you can try and get some laugh out of it from some point of view, saying ‘get off your bottom and go for a walk!’

FG6

However, participants also recognized that messages could be interpreted differently and the use of humor was risky, especially when participants were low in confidence.

...if I read that and I was in the wrong mood I’d take that as you’re telling me what to do, and I’d say ‘b*****r off.’

FG4

##### Motivational Texts

The feedback on motivational messages varied greatly. Overall, participants reported that the language and content of the motivational messages were acceptable due to the gentle, suggestive nature rather than “being told you’ve got to do it.” Some exemplar messages were perceived as a bit dated (eg, recommendations to not use a remote control to change the TV channel) or irrelevant (eg, tips about using stairs at home; “*...*but I live in a bungalow!”). Participants preferred practical tips and suggestions for increasing activity over more motivational suggestions (eg, “try writing down your barriers to activity this week”). In one focus group, participants suggested general supportive messages not necessarily linked to physical activity or health.

I know why I’m doing it [to reduce the chances of T2DM] so we don’t need reminding of it all the time.

FG7

##### Information Texts

The consensus was that messages focusing on health consequences of inactivity were too prominent and that a focus on benefits other than weight and reduced risk of T2DM would be preferred.

You could just say ‘good morning, this is PROPELS, hope you have a nice day’ or whatever...just simple—it doesn’t need to really say anything.

FG6

...when you’ve got a weight problem like I’ve got, I don’t need to be reminded—I’m doing my best!

FG4

##### Problem-Solving Texts

Some participants felt that the predefined response format was not appropriate for problem solving.

It’s like one of those PPI messages [spam text messages about reclaiming missold insurance]—I hate those!

FG4

However, others liked the idea that they could easily text in the reason why they had not achieved their goal. Participants generally liked the tailored and personalized texts that were triggered by responding to the problem-solving texts (eg, the message, “Take it easy this week. We hope that you feel better soon” as a response to selecting the illness or injury response option).

##### Tailoring

The concept of individually tailored text messages was very well received, especially in relation to individual goal progress and/or achievement.

You should get the one [text message] that’s relevant to you. If you’re doing more [steps], if you’re achieving your target or doing more, you still get one, but it should be different.

FG4

Participants advised that different people need different support, especially in terms of confidence and self-discipline in adhering to an activity plan. They suggested that messages should be less direct or less pushy if people are struggling to meet their goal and/or have mobility problems limiting the amount of walking that they could achieve.

##### Language and Frequency

We tested language variations within the messages. The general feedback was that the language needed to be formal, friendly, and polite with use of the participant’s name but limited use of emoticons.

I’m just warning you that it might be interpreted that you are shouting at us because in text language, capitals [letters] is shouting

FG5

It makes it sound as though you’re talking at us, rather than a computer.

FG6

Regarding the frequency of messages, participants responded that less is more. Overall, they perceived daily messages as too heavy-handed and potentially demotivating.

...otherwise if you are going to get this [text message] daily you’re going ‘oh another one’ and you get fed up with it.

FG6

In sum, the Phase 3 findings expanded the findings from the previous phases by (1) further emphasizing the importance of personalizing and tailoring messages according to key variables (eg, previous levels of physical activity, mobility issues that limit physical activity, individuals’ confidence in increasing physical activity, goal achievement/progress), (2) shaping the content of the messages (ie, the type of benefits to focus on within the motivational messages), (3) informing the frequency of messages and sequencing of the follow-on support program, and (4) highlighting the importance of including other activities (eg, cycling, swimming) to maintain engagement of participants who did other activities than walking alone.

As a result of the findings from Phases 2 and 3, we added a Week 1 educator telephone call (Figure 4): a brief telephone-administered assessment including key information required to tailor subsequent text messages. We also added a conversion chart to the activity diary, which would enable participants to convert other activities (for which they might not be wearing their pedometer or for which they perceive a pedometer to not accurately assess) into steps for texting in. For example, this chart includes descriptions of other activities (such as swimming breaststroke, moderate effort, and cycling 10 mph) and provides a conversion into a step count, based on MET equivalents [50], that can be added to the participant’s total.

### Phase 4: Piloting

In the final piloting phase, we developed a full set of text messages and tailoring matrices for the initial 8 weeks of the follow-on support program. Examples of the tailoring matrices for Weeks 1 and 4 are shown in Multimedia Appendix 2. In this section, we present findings on the participant feedback on the content and structure of the program followed by technical issues.

#### Program Content and Structure

Most participants found that the follow-on support motivated them to be physically active due to increased awareness of their own activity. Participants found the telephone call, in which the brief assessment was administered, helpful in providing additional support, especially with overcoming any technical barriers.

But, you've got somebody there you can speak to then say, “right I'm having a problem, I've done such and such and I can’t register me steps” or whatever, it's just saying, “right, you should do this or I’ll get somebody to ring you back and tell you what to do,” you can't do that on text can you?

R5

Participants reported that the system provided continued support and encouragement. For example, the reminder texts were helpful prompts to continue self-monitoring; continued goal setting and immediate feedback provided further motivation to be active.

It’s quite nice. It keeps me sort of in the zone in the fact that I enjoy using the pedometer because it keeps my mind on exercise. I’m conscious of it, and, you know, if I haven’t done too much moving about, I go and walk some more.

R5

I usually do remember to put me pedometer on...but as I say it’s nice to know there’s a reminder there and when I send off my figures I get an immediate response. I think it’s all been quite encouraging actually.

R4

They reported that the frequency of messages (at most 2 per week) to be sufficient for the 8-week period but commented that over time the messages could decrease in frequency as they would not need as much reminding.

As I say I think at the beginning you need more frequent reminders, you know I think you’ve got that right, and then as it goes on you don’t need so many.

R6

Overall, participants were positive about the text message content, readability, and clarity and struggled to recall examples of discouraging messages. Several participants picked out the feedback texts and motivational texts, which provided instructions (tips) for increasing physical activity, as particularly useful.

Do you know I’ve even started...this is what you have got me doing...when I’m on the kitchen chair, making a cup of coffee or something, I start running on the spot for a hundred! I count up to a hundred, running on the spot. So that’s another hundred steps!

R10

Those who did not consistently increase their step counts reported receiving slightly more negative messages but none they perceived as chastising.

I found that very encouraging. It was good. When I’d done a good week, it’s very...I only missed one week, and although you didn’t down me, you didn’t say anything nasty, you just said try a little harder, I know it’s hard to get the exercise in, so I found it very encouraging.

R1

#### Technical Issues

A total of 9 of the 11 participants received the full regimen of text messages as intended. Minor technical glitches impeded the full delivery to 2 participants. Most participants had no difficulty registering with the text system, and more than 90% of all incoming messages from the participants were correctly formatted. Almost all participants responded to at least two prompting texts and received tailored feedback on at least two occasions. Three-quarters responded to all prompting texts and received tailored feedback texts every week.

Several participants were unclear about the type of messages they could respond to. Some sent thank you messages in response to the feedback texts and then received a text message about unrecognized format.

I was just replying to your request or your advice, when I didn’t do the correct steps one week, you gave me a couple of bits of helpful advice and I text back thanking you for that, and obviously it wouldn’t let me send.

R7

Related to this, participants wanted a greater degree of flexibility in the format for texting in step counts. They were asked to enter the word “steps” followed by their weekly step total but some submitted only numbers or the word “step” or “step-count for week,” which triggered an unrecognized response text.

Finally, participants with limited experience in texting reported receiving and reading texts without problem but utilized help from relatives (usually grandchildren) when prompted to text in their weekly step counts.

Oh, yes, I could [read all the messages]...it’s just getting them sent off. Because again I think this week I was late, I thought I’d sent them in twice and then I had to check with [granddaughter], and I think I had pressed some other button. I think I’ve got a handle on it now. It sounds stupid but they didn’t have all these phones back then.

R2

In sum, the piloting phase indicated that (1) the structure of the follow-on support (including the brief telephone call) was acceptable, (2) the frequency of text messages over the 8-week pilot phase was acceptable but should be reduced over time, (3) the content and language used in the text messages were acceptable, (4) minor technical issues needed to be resolved, and (5) participant instructions in both the Walking Away session and the follow-on support booklet required refinement.

#### The Final PROPELS Follow-On Support Program

The findings from each phase were consolidated into a finalized set of text messages and underpinning schedule with integrated tailoring. This involved the development of tailoring matrices for each week of the program with additional messages for Years 2 through 4 (to ensure that there was sufficient variation in message content for the 4-year study). We briefly describe each component of the resulting PROPELS RCT follow-on support program in Figure 4.

## Discussion

### Principal Findings

Using a systematic approach to the development and piloting of the PROPELS text-messaging and pedometer follow-on support program, we identified the following key components: differing frequency of text messages according to period and year of program, tailored text message content according to key variables, personalized text messages using the participant’s nickname, facility for two-way interaction, use of motivational texts emphasizing affective benefits rather than health benefits, and inclusion of general encouragement messages. Participant need for social support from and rapport with the educator and the need for a way of eliciting information for tailoring resulted in the addition of supplemental telephone calls. Furthermore, we identified and addressed potential barriers such as impersonal messages or unfamiliar technology.

A key task was to assess the acceptability of a text-messaging intervention for our target group: older adults at risk of T2DM. Their active involvement in the intervention development phases resulted in specific components to meet their needs. For example, an instruction booklet about the text-messaging program and telephone calls to supplement the text messages were added to the follow-on support program to facilitate user engagement. We acknowledge that some initial training or help with text messaging (at the initial Walking Away education session) may be required to ensure that all participants are able to engage with this type of text-messaging support. Automated tailored text messaging following structured group education enables initial one-to-one help with getting started and reduces the time commitment for health care professionals and participants. It is scalable and fits into participants’ everyday lives while maintaining ongoing support following the initial education session. This is particularly important in primary care, where many people are identified as being at risk through health checks (eg, NHS Health Check, in England [51]) but where there is limited capacity for providing ongoing support for behavior change. In England, a key objective of the NHS Five-Year Forward View [52] is to implement scalable diabetes prevention programs; if successful, the PROPELS intervention may be an ideal candidate for this. Future research could explore variations of follow-on support: providing the follow-on support as a standalone intervention or pairing the follow-on support with a one-off telephone call that covers the Walking Away education session content for people who are unable or unwilling to attend group-based structured education.

The PROPELS text-messaging and pedometer follow-on support could be adapted fairly easily for other target groups such as people with newly diagnosed or established T2DM attending structured education (eg, DESMOND [Diabetes Education and Self-Management for Ongoing and Newly Diagnosed] [53]) or people with or at risk of other conditions (eg, cardiovascular disease) where increasing physical activity reduces the risk of developing the condition or its consequences.

### Limitations

Time constraints related to timelines of the PROPELS RCT [30] meant that we were unable to conduct a pilot of longer duration to test the acceptability of varying text-messaging frequency, participant engagement, and retention over time. These are assessed in the PROPELS RCT along with physical activity outcomes [30]. Further qualitative work embedded within the RCT may identify potential future adaptations and facilitate long-term implementation and could provide an in-depth understanding of how participants engage with the program over time, which components are most and least helpful, and how pedometer use, text messages, and telephone calls influence physical activity change over time.

We acknowledge that, especially in developed countries, text messaging may become less acceptable over time, and participants may prefer newer technologies such as smartphones that incorporate accelerometers. Although older adults have relatively low rates of smartphone ownership [18], recent research indicates that text messaging is becoming increasingly popular with that age group [54]. Taken together, this indicates that mHealth interventions through smartphones would have had limited reach in the PROPELS study, and a predominantly text message-focused program is currently more acceptable. One advantage (and direction for future work) of the PROPELS follow-on support program is that it could be easily adapted for delivery across a variety of platforms (eg, email, app) which would allow people to choose which version they use.

A final potential limitation relates to the weekly reporting of steps. Although PROPELS participants are encouraged to record their daily step count in their activity diary and text in the weekly total, there is more room for error in comparison to, for example, texting in each day’s total in response to a daily prompt. However, our participants voiced aversion to the idea of daily texts as overkill and off-putting. Future qualitative work in the PROPELS trial may provide an insight into participant experiences and preferences for self-monitoring step counts.

### Comparisons With Prior Work

We developed a novel, interactive program whereby participants self-monitor their physical activity using a pedometer, text in their weekly step count, and receive automated tailored feedback on goal achievement and progress. Previous mHealth interventions for T2DM prevention included untailored, passive text-messaging content such as information about T2DM risk [20].

The methods that we employed to develop the PROPELS follow-on support program combine features of published mHealth development frameworks [31,32] and multiple iterative phases of qualitative research (similar to a user-centered design process [55]). The high level of engagement with our target population enabled refinements in the design to optimize its acceptability to users.

Robust development of mHealth behavior change interventions can be time consuming [33,56] and is often allocated limited time in RCT protocols. A potential consequence of rapid development is that insufficient attention is given to the underpinning theory and evidence base or selection of active ingredients (BCTs). Given the time constraints of the PROPELS RCT protocol (12 months to conceptualize, develop, and test the follow-on support program prior to the RCT’s commencement), this paper provides a detailed outline of a pragmatic framework for developing and piloting a text-messaging intervention that draws on relevant behavior change theory and uses rigorous qualitative methods incorporating user engagement. It encourages replication and application to the development of similar interventions.

### Conclusions

We developed a feasible and innovative text-messaging and pedometer program based on evidence and behavior change theory and grounded in the experiences, views, and needs of people at high risk of T2DM. A large-scale RCT is testing the effectiveness of this 4-year program over and above group-based structured education alone.

## Appendix

Multimedia Appendix 1 Key findings from Phase 1: Intervention objectives, determinants of physical activity targeted in the PROPELS follow-on support program, and included behavior change techniques.

Multimedia Appendix 2 Example tailoring matrices.

## Abbreviations

BCT: behavior change technique
IGR: impaired glucose regulation
NHS: National Health Service
NIHR: National Institute for Health Research
PROPELS: Promotion of Physical activity through Structured Education with differing Levels of ongoing Support for people at high risk of type 2 diabetes
RCT: randomized controlled trial
T2DM: type 2 diabetes
