# Supplementary material for: A Text-Messaging and Pedometer Program to Promote Physical Activity in People at High Risk of Type 2 Diabetes: The Development of the PROPELS Follow-On Support Program
Source: JMIR Mhealth Uhealth. 2015 Dec 15;3(4):e105. doi: 10.2196/mhealth.5026 (PMC4704921; doi:10.2196/mhealth.5026)
Supplement: Multimedia Appendix 1 [file mhealth_v3i4e105_app1.pdf]

Appendix 1: Key findings from Phase 1–Intervention objectives, determinants of physical activity targeted in the PROPELS follow-on support programme, and included behaviour change techniques.

Intervention objectives of the PROPELS follow-on support programme

Primary objectives:	Enhance the use of self-regulatory strategies to increase PA.
Secondary objectives:	Make the benefits of PA personally relevant (positive attitudes and beliefs).<br>Ensure that participants are motivated to increase and maintain their PA.<br>Enhance positive outcome expectancies and self-efficacy in relation to PA.

**Determinants of behaviour change**	**How will we achieve this?**	**BCTs included (in final programme)**	**Intervention component in which this BCT occurs**	
			Follow-on support	
			Text-messages	Phone call
1. Self-regulatory strategies	1.1 Encourage participants to set short- and long-term PA goals.	1. Goal setting (behaviour)		X
		2. Action planning	X	X
	1.2 Encourage participants to develop action plans (and reinforce these over the course of the follow-on support programme).	3. Self-monitoring of behaviour	X	
		4. Feedback on behaviour	X	X
		5. Discrepancy between current behaviour and goal	X	X
	1.3 Encourage and reinforce self-monitoring (wearing the pedometer and	6. Review behaviour goals		X
		7. Problem solving	X	X

	logging steps).<br>1.4 Provide feedback relating to goal achievement and progress.<br>1.5 Highlight the discrepancy between current behaviour and goals.<br>1.6 Review initial PA goals and amend these accordingly.<br>1.7 Help participants to overcome barriers to increasing their PA		
2. Attitudes and beliefs	2.1 Reinforce health, emotional and social benefits of increasing PA (e.g., persuade that increasing PA will make participants feel better generally and also reduce risk of T2DM).<br>2.2 Highlight that by increasing PA by even a small amount can	1a. Information about health consequences	X
		1b. Information about social consequences	X
		2. (Re)framing	X
		3. Pros and cons	X

	have numerous health benefits.<br>2.3 Encourage participants to reflect on the benefits and negatives of changing their behaviour			
3. Motivation	3.1 Provide positive reinforcement regarding progress and achievement of PA-related goals.<br>3.2 Encourage plans to reward oneself (appropriately) if goals are achieved.<br>3.3. Encourage PA with others to stay motivated.<br>3.4 Encourage alternative activities to help participants stay motivated to meet their step goals.<br>3.5 Promote positive self-talk.<br>3.6 Encourage the use	1. Social reward	X	
		2. Self-incentive	X	
		3. Social support (practical)	X	X
		4. Instruction on how to perform the behaviour	X	
		5. Self-talk	X	
		6. Prompts/cues	X	
		7. Habit formation	X	
		8. Commitment	X	

	of visual cues and prompts.<br>3.7 Encourage ways of enabling PA to become habitual in participants’ lives.<br>3.8 Encourage participants to make a commitment to increasing (or maintaining) their activity.			
4. Self-efficacy and Outcome expectancies	4.1 Ensure participants feel supported.<br>4.2 Help participants believe that they are able to overcome barriers in order to stick to action plans and achieve goals.<br>4.3 Help participants to focus on past success if previously physically active.<br>4.4 Promote imagining of future outcomes	1. Social support (emotional)	X	X
		2. Verbal persuasion about capability	X	
		3. Focus on past success	X	
		4. Comparative imagining of future outcomes	X	

(being active versus
inactive)
